# Sport neuroscience revisited (?): a commentary

**DOI:** 10.3389/fnhum.2014.00929

**Published:** 2014-12-05

**Authors:** Stergios Makris

**Affiliations:** Department of Psychology, Edge Hill UniversityOrmskirk, UK

**Keywords:** sport, neuroscience, application, action, visual perception

Professor Vincent Walsh in his recently published review (Walsh, 2014) has described sport and the performance of elite athletes as an excellent opportunity for Cognitive Neuroscience to overcome the barrier between lab-based research and applicability in the real world. Indeed, it is a worrying phenomenon that the radical development of cognitive neuroscience in the last few years has only been accompanied by unfulfilled promises for applications to real world domains, such as education and psychiatry. On the other hand, research within sport science and its fostered child, sport psychology, keeps providing us with strong evidence that elite athletes have extraordinary abilities covering a whole range of behaviors, from managing stress and fatigue to superior action performance (i.e., Aglioti et al., 2008).

In the sport domain, individuals exhibit a great amount of motor expertise, accompanied by superior perceptual abilities. Extensive research with motor experts in the past, such as professional ballet dancers, has shown a link between executing actions and understanding observed actions by others (Calvo-Merino et al., 2006; Cross et al., 2006). However, it has been only due to research with athletes that we managed to achieve a better understanding of this common neural network between motor performance and action perception. It has been suggested that elite athletes own a unique ability to perceive body kinematics and simulate observed actions in sport sequences that they are familiar with (Abernethy et al., 2008; Aglioti et al., 2008; Urgesi et al., 2012). Moreover, it has been indicated that the bigger that familiarity with observed actions is, the better their predictions will be for the future of observed action sequences; thus, suggesting an internal mechanism for simulating observed actions depending on motor expertise and familiarity.

Research findings of this kind have drawn new interest in the neural correlates of superior action prediction abilities in athletes, yearning at the same time to apply these data in the day-to-day physical preparation and training. A recent study, for example, by Tomeo et al. (2013) has tried to investigate the perceptuo-motor processes of the soccer players' ability to identify deceptive actions by their opponents in the field. Indeed, the authors have presented strong evidence that athletes, as compared to novices, are far better into predicting fooling soccer actions and that the ability to successfully respond to deception lies on the constant update of simulative motor representations of the opponents' actions. In a more recent study, Makris and Urgesi (2014) have further investigated the neural underpinnings of deceptive actions prediction in the soccer domain. In an experiment trying to identify the specific roles of motor, premotor, and visual areas in the simulation of soccer action sequences (i.e., penalty kicks) with or without deception, the authors have managed to obtain, for the first time, causative evidence by means of transcranial magnetic stimulation (TMS) techniques of the complimentary functional roles of visual (superior temporal sulcus; STS) and premotor (dorsal premotor cortex; PMd) areas in athletes' action perception and prediction skills.

Even though someone could argue that the aforementioned data are still purely experimental and thus constrained to the lab environment, we have to consider that elite sport performance is a constant struggle of at least one or two decades in the life of an athlete. During this time their brains are transforming into a superior organ, able to support and maintain the enactment of their sport abilities. It would be erroneous, therefore, if we tried to dissociate an athlete's performance in the field from that in a lab-based setting. After all, a considerable amount of sport physical preparation and training takes place nowadays in special venues and with technical equipment that altogether do not necessarily reflect the environment of a sport field. In that sense, we see no reason why neurophysiological data obtained by athletes should stay in the lab (or the computer desktop “recycle bin” of a journal's editor!) and not disseminated to those directly involved; the athletes themselves, their coaches, physical trainers and sport clubs in general.

So far, there have been only a few cases reported of neuroscience research directly applied to sport practice. For example researchers from the Korea University College of Medicine have tried to investigate whether neuroplasticity is sport-specific (Park et al., 2012). For this, they used speed-skating athletes that during sport execution they have to run counter-clockwise around the track. The researchers inferred that years of practice to perfect one-direction movement must show a unique growth pattern in the brain not else observed; and indeed neuroimaging data has revealed that the right hemisphere of these athletes' cerebellum was more developed that the opposite one, as an outcome of them always standing on their right foot during sport performance. On the other side of the Atlantic, neuroscience researchers from Columbia University (Young-Rojahn, 2013) are currently working on a US government-funded project to investigate the unique brains of basketball players and answer questions, such as how skill can be used to deal with stress and how “court vision” is represented in the brain and thus can be trained (see also Biomedicine News, March 2013). Finally, quite recently cognitive neuroscientists from around the world proposed a new methodological approach for acquiring neurophysiological data without the need of a lab establishment. They have called it “mobile brain/body imaging (MoBI)” and they describe it as a novel and radical approach for overcoming the lab barriers and collecting data in the field during real-time performance (Gramann et al., 2014).

Overall, cognitive neuroscience has the tools and resources today to overcome its lab barriers and show to the world the real potential and impact of our work. And this is not just by publishing in highly-specific scientific journals or presenting in peer-packed conferences. In cases like sport, we owe our experimental work to all the volunteers that signed our forms, agreed to sit in our dim-light labs and do our “not-making-sense” (most of the time) tasks. We may need to, also, recall the passion with which they did that. With the same passion we need to get back to them and live up to our promises. The game is on. Let's play it in the right field this time.

## Conflict of interest statement

The author declares that the research was conducted in the absence of any commercial or financial relationships that could be construed as a potential conflict of interest.
